# Supplementary material for: Exploring the Interplay Between Healthcare Quality and Economic Viability Through Massive Data Analysis-Driven Multi-Hospital Management in a Spanish Private Multi-Hospital Network
Source: Healthcare (Basel). 2025 Nov 24;13(23):3034. doi: 10.3390/healthcare13233034 (PMC12692472; doi:10.3390/healthcare13233034)
Supplement: Supplementary file 1 [file healthcare-13-03034-s001.zip › Supplementary Figure S1.pdf]

Supplementary Figure S1. Correlation matrix of the initial set of KPIs.

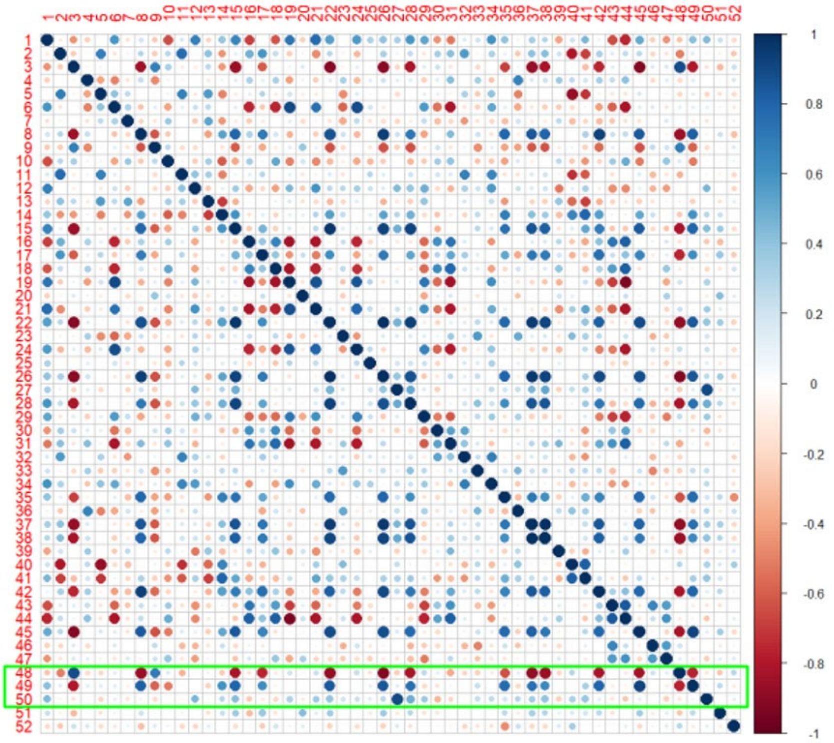

Figure S1. The matrix illustrates the linear relationships between the 52 initial KPIs. Colors indicate the magnitude and direction of correlations (blue = positive, red = negative), while the size of the points reflects correlation strength. As expected, the diagonal shows values of 1, corresponding to self-correlation. A cluster of highly correlated variables (|r| > 0.90), particularly involving KPIs 48 to 50, was identified. Three indicators were excluded from the dataset to avoid redundancy and potential multicollinearity in the subsequent analyses.

Indexes for the correlation matrix:

1. Percentage of patients discharged before noon
2. Percentage of ER patients referred to outpatient clinics
3. Percentage of urgent surgeries
4. Percentage of compliance with Surgical Checklist
5. Percentage of patients admitted through ER
6. Percentage of interventions with complete anesthesia informed consent
7. Percentage of interventions with complete surgical informed consent
8. Operating Room occupancy percentage
9. Percentage of new patients in outpatient clinics
10. Percentage of first consultations over total consultations
11. Percentage of ER patients attended within 30 minutes
12. Percentage of ER treatments completed within 90 minutes
13. Percentage of triaged ER patients
14. Total discharges from non-surgical episodes
15. Total discharges from surgical episodes
16. Standard ratio of major outpatient surgery over total surgeries
17. Standard complexity ratio of procedures or cases in hospitalization and major outpatient surgery
18. Inverse of Standard Ratio of complications in hospitalizations or major outpatient surgery
19. Inverse of Average wait time for first medical consultation
20. Inverse of Standard Ratio of average hospital stay duration
21. Percentage of hospitalization episodes with all required nursing evaluations
22. Total available operating room hours
23. Percentage of discharge reports completed according to defined procedures

24. Standard intensity ratio of coded diagnoses in clinical report or major outpatient surgery
25. Inverse of Standard Ratio of mortality
26. Total number of surgeries performed
27. Total number of births attended
28. Number of high complexity techniques performed in OR
29. Percentage of surgical reports completed according to defined procedures
30. Net Promoter Score for outpatient consultations
31. Net Promoter Score for major outpatient surgery
32. Global Net Promoter Score of the hospital
33. Net Promoter Score for hospitalization
34. Net Promoter Score for ER
35. Percentage of occupied beds in the hospital
36. Percentage of medication orders completed according to defined procedures
37. Number of first visits to medical specialties
38. Number of first visits to surgical specialties
39. Inverse of Standard Ratio of readmitted patients
40. Number of ER cases attended in adult patients
41. Number of ER cases attended in pediatric patients
42. Value of generated sales
43. Value of sales generated in the three months following
44. Value of sales generated in the six months following
45. Profit margin
46. Profit margin in the three months following
47. Profit margin in the six months following
48. Percentage of patients discharged from ER
49. Labor days
50. Number of cesarean deliveries
51. Average hospital stay duration pre-surgery
52. Standard Rate of Potentially Avoidable Hospitalizations

The excluded variables from the analysis, as well as their maximum degree of correlation with any of the other variables in the study, are detailed below:

48. Percentage of patients discharged from ER → -0.91 (with total number of surgeries performed [Nº 26])
49. Labor Days → 0.91 (with profit margin [Nº 45])
50. Number of cesarean deliveries → 0.90 (with total number of births attended [Nº 27])

Additionally, the outliers for indicator 51, which were not compatible with the rest of the data, were removed from the study, as was the case with the previous three indicators. Below, a box plot is presented showing the analysis of the distribution.
