# Supplementary material for: Exploring the Interplay Between Healthcare Quality and Economic Viability Through Massive Data Analysis-Driven Multi-Hospital Management in a Spanish Private Multi-Hospital Network
Source: Healthcare (Basel). 2025 Nov 24;13(23):3034. doi: 10.3390/healthcare13233034 (PMC12692472; doi:10.3390/healthcare13233034)
Supplement: Supplementary file 1 [file healthcare-13-03034-s001.zip › Supplementary Figure S2.pdf]

Supplementary Figure S2. Hospital homogeneity dendrogram.

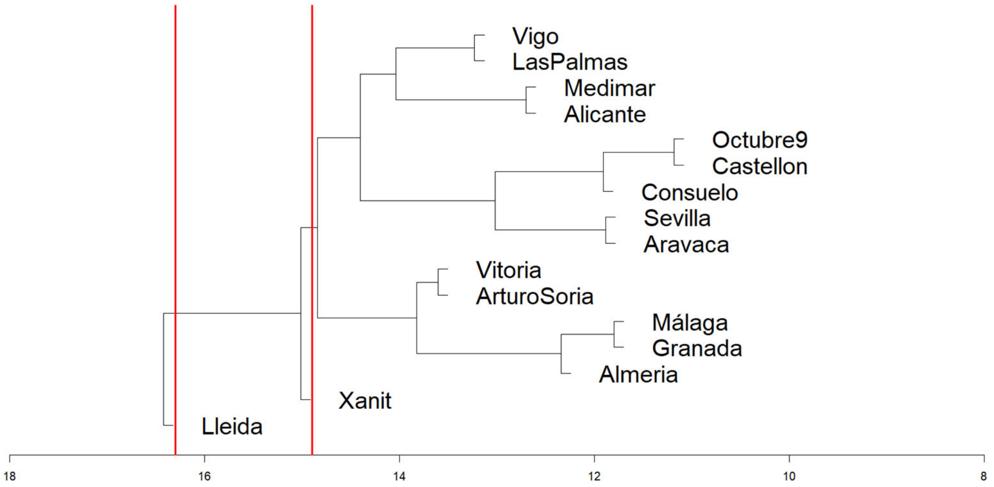

Figure S2. Hierarchical clustering of the 16 hospitals using Ward's method and Euclidean distance. The dendrogram shows two centers (Vithas Lleida and Vithas Xanit) with markedly distinct profiles compared to the rest of the network. To ensure dataset homogeneity and avoid bias in the subsequent analyses, these two hospitals were excluded from the final sample.
