# Supplementary material for: Exploring the Interplay Between Healthcare Quality and Economic Viability Through Massive Data Analysis-Driven Multi-Hospital Management in a Spanish Private Multi-Hospital Network
Source: Healthcare (Basel). 2025 Nov 24;13(23):3034. doi: 10.3390/healthcare13233034 (PMC12692472; doi:10.3390/healthcare13233034)
Supplement: Supplementary file 1 [file healthcare-13-03034-s001.zip › Supplementary Figure S3.pdf]

Supplementary Figure S3. **Comparative assessment of artificial intelligence models.**

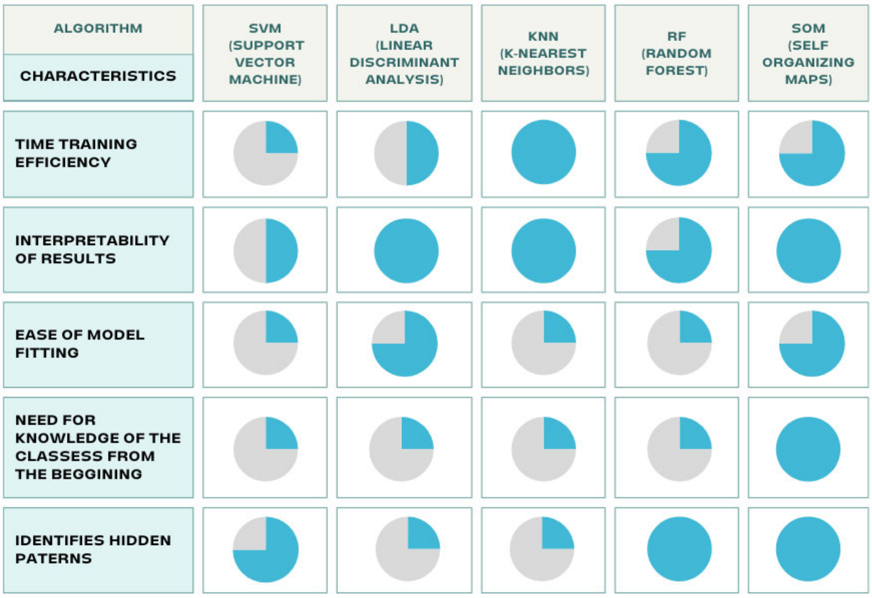

Figure S3. Qualitative evaluation of five algorithms—Support Vector Machines (SVM), Linear Discriminant Analysis (LDA), k-Nearest Neighbors (k-NN), Random Forest (RF), and Self-Organizing Maps (SOM)—across five predefined criteria: training efficiency, interpretability of results, ease of model fitting, requirement for prior class knowledge, and ability to identify hidden patterns. Ratings are shown on a three-level scale (empty = low, half = moderate, full = high). SOM demonstrated the most balanced performance across all criteria, providing the rationale for its selection as the core analytical tool in this study.

Comparative assessment of five artificial intelligence models (SVM, LDA, k-NN, RF, and SOM) according to predefined criteria: training efficiency, interpretability of results, ease of model fitting, requirement for prior class knowledge, and ability to identify hidden patterns. Rating scale: empty = low, half = moderate, full = high.
