# Supplementary material for: Exploring the Interplay Between Healthcare Quality and Economic Viability Through Massive Data Analysis-Driven Multi-Hospital Management in a Spanish Private Multi-Hospital Network
Source: Healthcare (Basel). 2025 Nov 24;13(23):3034. doi: 10.3390/healthcare13233034 (PMC12692472; doi:10.3390/healthcare13233034)
Supplement: Supplementary file 1 [file healthcare-13-03034-s001.zip › Supplementary Figure S4.pdf]

Supplementary Figure S4. Adjustments to the configuration parameters.

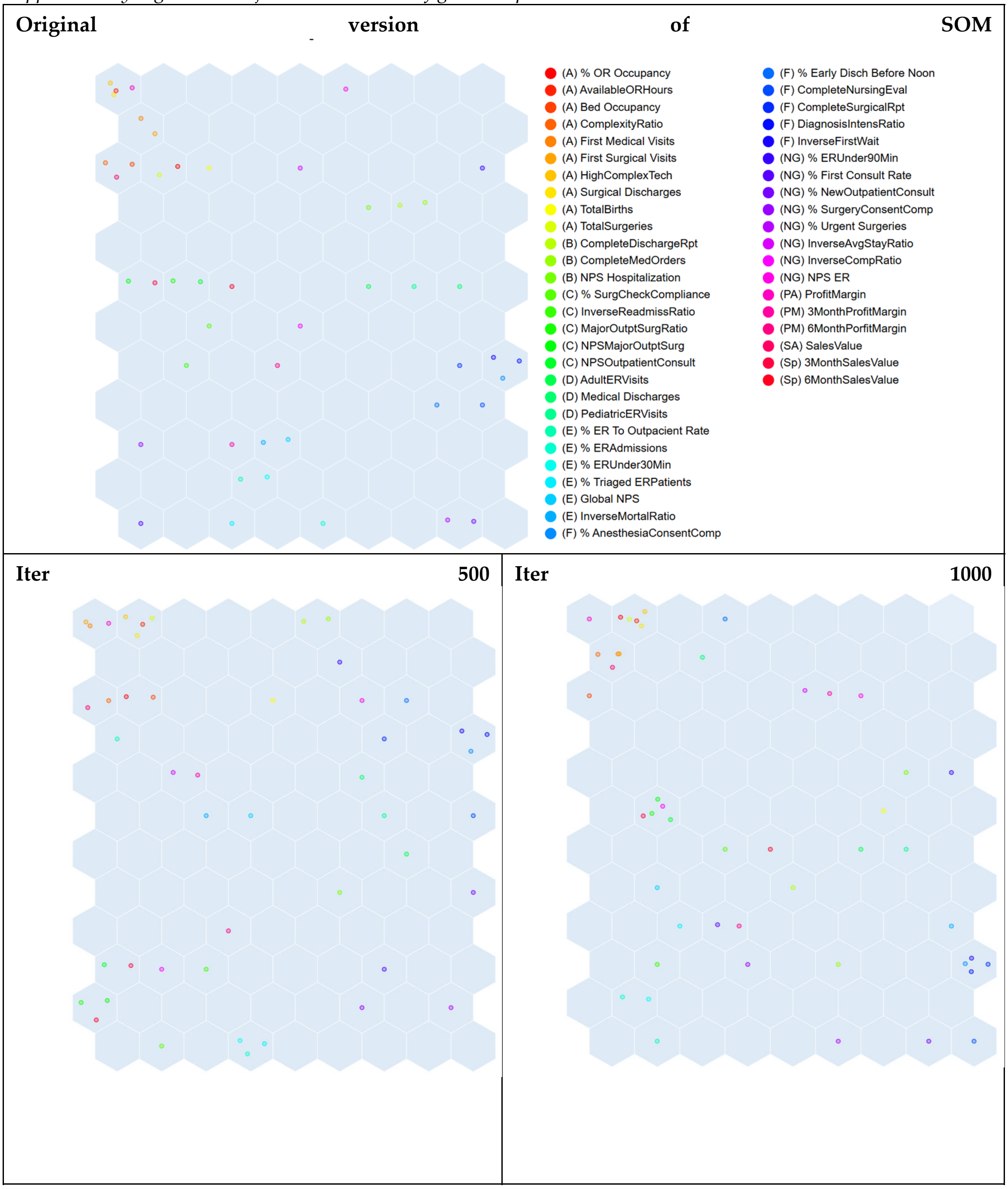

Figure S4. Adjustments to the configuration parameters. The figure presents a comparison of data point distribution within a hexagonal grid at two different iterations: Iter 500 (left) and Iter 1000 (right).
