# Supplementary material for: Exploring the Interplay Between Healthcare Quality and Economic Viability Through Massive Data Analysis-Driven Multi-Hospital Management in a Spanish Private Multi-Hospital Network
Source: Healthcare (Basel). 2025 Nov 24;13(23):3034. doi: 10.3390/healthcare13233034 (PMC12692472; doi:10.3390/healthcare13233034)
Supplement: Supplementary file 1 [file healthcare-13-03034-s001.zip › Supplementary Figure S6.pdf]

Supplementary Figure S6. *Resampling*

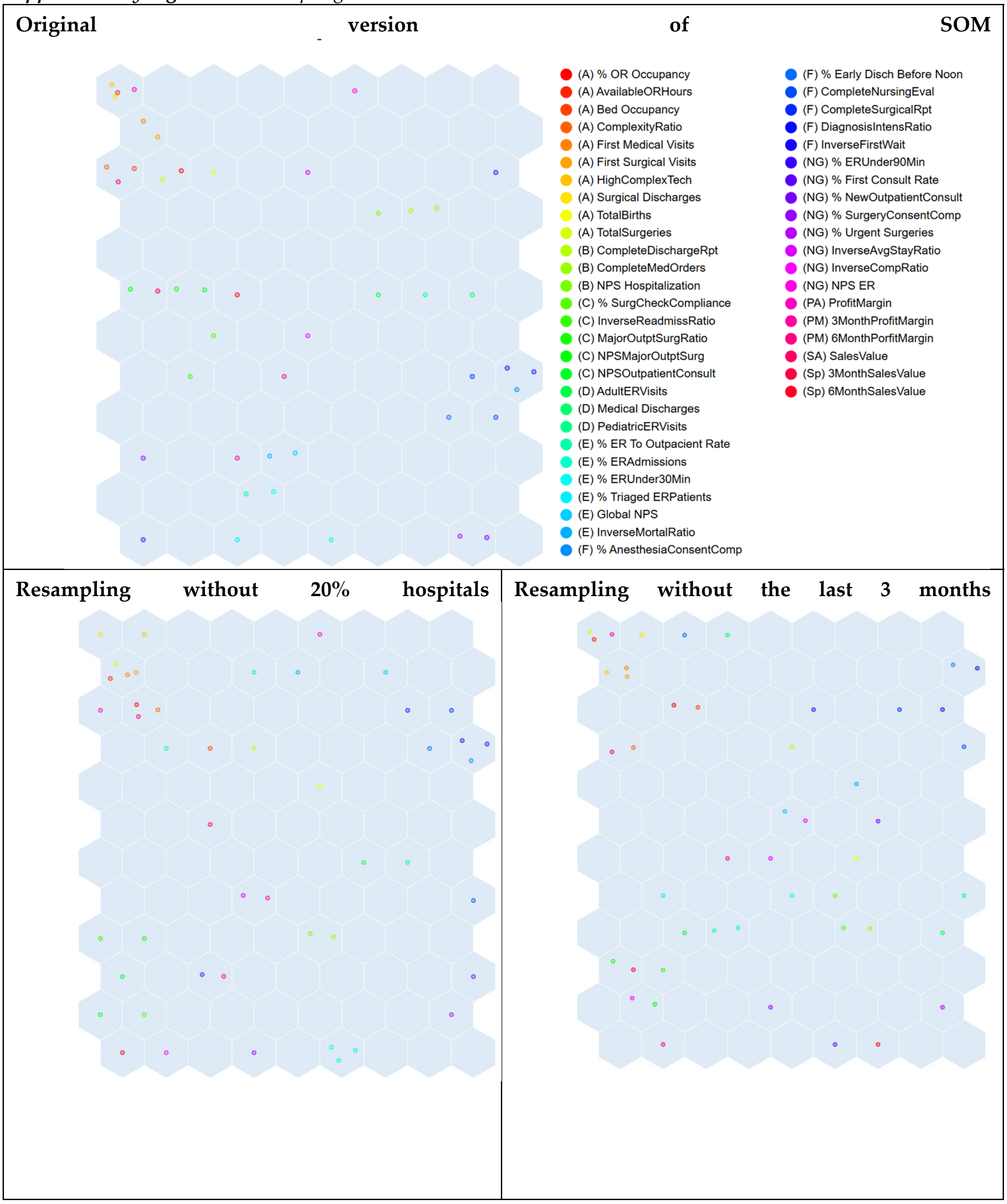

Figure S6. Resampling. The figure compares the distribution of data points within a hexagonal grid under two different resampling scenarios: Resampling 20% of hospitals (left) and Temporal resampling (without the last period) (right).
