# Supplementary material for: Exploring the Interplay Between Healthcare Quality and Economic Viability Through Massive Data Analysis-Driven Multi-Hospital Management in a Spanish Private Multi-Hospital Network
Source: Healthcare (Basel). 2025 Nov 24;13(23):3034. doi: 10.3390/healthcare13233034 (PMC12692472; doi:10.3390/healthcare13233034)
Supplement: Supplementary file 1 [file healthcare-13-03034-s001.zip › Supplementary Figure S7.pdf]

Supplementary Figure S7. *Outiers*

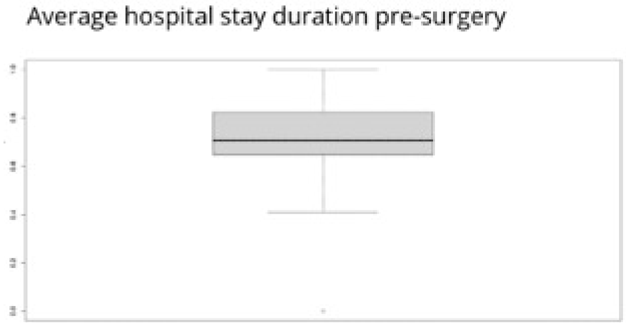

Figure S7. The box plot illustrates the average duration of hospital stay before surgery, displaying the median, data dispersion, and the presence of outliers, which are represented as points beyond the interquartile range limits.
