# Supplementary material for: Exploring the Interplay Between Healthcare Quality and Economic Viability Through Massive Data Analysis-Driven Multi-Hospital Management in a Spanish Private Multi-Hospital Network
Source: Healthcare (Basel). 2025 Nov 24;13(23):3034. doi: 10.3390/healthcare13233034 (PMC12692472; doi:10.3390/healthcare13233034)
Supplement: Supplementary file 1 [file healthcare-13-03034-s001.zip › Supplementary Table S1.pdf]

Supplementary Table S1 . KPIs details. KPIs included in the study. The following table shows the 47 KPIs ultimately used in the analysis, along with their abbreviations

**KPIs name**	**Abbreviation**	**Abbreviation Shown In SOM**
Percentage of patients discharged before noon	Early Discharges	% Early Disch. Before Noon
Percentage of ER patients referred to outpatient clinics	ER to Outpatient Rate	% ER To Outpatient Rate
Percentage of urgent surgeries	Urgent Surgeries	% Urgent Surgeries
Percentage of patients admitted through ER	ER Admissions	% ER to Hosp. Admissions
Operating Room occupancy percentage	OR Occupancy	% OR Occupancy
Percentage of new patients in outpatient clinics	New Outpatient Consultations	New Patients Outp. Consult.
Percentage of first consultations over total consultations	First Consultations Rate	% First Consult Rate
Percentage of ER patients attended within 30 minutes	ER Under 30 Min	% ER Under 30 Min.
Percentage of ER treatments completed within 90 minutes	ER Under 90 Min	% ER Under 90 Min.
Percentage of triaged ER patients	Triaged ER Patients	% Triaged ER Patients
Discharges from non-surgical episodes	Medical Hospital Discharges	Medical Hosp. Discharges
Discharges from surgical episodes	Surgical Hospital Discharges	Surgical Hops. Discharges
Standard ratio of major outpatient surgery over total surgeries	Major Outpatient Surgery Ratio	Outpatient Surg. Ratio
Standard complexity ratio of procedures or cases in hospitalization and major outpatient surgery	Complexity Ratio	Complexity Ratio
Ebitda	Ebitda (Current Month)	Ebitda (Current Month)
Total available operating room hours	Available OR Hours	Available OR Hours
Standard intensity ratio of coded diagnoses in clinical report or major outpatient surgery	Diagnosis Intensity Ratio	Diagnosis Intens. Ratio
Total number of surgeries performed	Total Surgeries	Total Surgeries
Total number of births attended	Total Births	Total Births
Number of high complexity techniques performed in OR	High Complexity Techniques	High Complex. Proced.
Net Promoter Score for outpatient consultations	NPS Outpatient Consultations	NPS Outpatient Consult.
Net Promoter Score for major outpatient surgery	NPS Major Outpatient Surgery	NPS Outpatient Surg.
Global Net Promoter Score of the hospital	Global NPS	Global NPS
Net Promoter Score for hospitalization	NPS Hospitalization	NPS Hospitalization

Net Promoter Score for ER	NPS ER	NPS ER
Percentage of occupied beds in the hospital	Bed Occupancy	Bed Occupancy
Number of first visits to medical specialties	First Medical Visits	First Medical Visits
Number of first visits to surgical specialties	First Surgical Visits	First Surgical Visits
Number of ER cases attended in adult patients	Adult ER Visits	Adult ER Visits
Number of ER cases attended in pediatric patients	Pediatric ER Visits	Pediatric ER Visits
Value of generated sales	Sales (Current Month)	Sales (Current Month)
Percentage of compliance with Surgical Checklist	Surgical Checklist Compliance	% Surg. Check Compliance
Percentage of interventions with complete anesthesia informed consent	Anesthesia Consent Compliance	% Anesthesia Consent Comp.
Percentage of interventions with complete surgical informed consent	Surgery Consent Compliance	% Surgery Consent Comp.
Ebitda generated in the three months following	Ebitda (Current Month +3)	Ebitda (Current Month+3)
Ebitda generated in the six months following	Ebitda (Current Month +6)	Ebitda (Current Month+6)
Percentage of hospitalization episodes with all required nursing evaluations	Complete Nursing Evaluation	Complete Nursing Eval.
Percentage of discharge reports completed according to defined procedures	Complete Discharge Report	Complete Discharge Rpt.
Percentage of surgical reports completed according to defined procedures	Complete Surgical Report	Complete Surgical Rpt.
Percentage of medication orders completed according to defined procedures	Complete Medication Orders	Complete Med. Orders
Value of sales generated in the three months following	Sales (Current Month+3)	Sales (Current Month+3)
Value of sales generated in the six months following	Sales (Current Month+6)	Sales (Current Month+6)
Inverse of Standard Ratio of complications in hospitalizations or major outpatient surgery	Inverse Complications Ratio	Complication Ratio (Inv.)
Inverse of Average wait time for first medical consultation	Inverse First Consultation Wait	First Consult Wait (Inv.)
Inverse of Standard Ratio of average hospital stay duration	Inverse Average Stay Ratio	Avg. Stay Ratio (Inv.)

Inverse of Standard Ratio of mortality	Inverse Mortality Ratio	Mortal. Ratio (Inv.)
Inverse of Standard Ratio of readmitted patients	Inverse Readmission Ratio	Readmiss. Ratio (Inv.)

Supplementary Table S1 The following table presents the clinical, healthcare and financial indicators used in the study, together with their basic descriptive statistics in order to provide an overview of the distribution of the data analyzed.

KPI	n	Mean	Median	STD	Min	Q1	Q3	IQR	Max	Kurtosis
% patients discharged before noon	594	0.5286350	0.529792	0.2662083	0	0.32453846	0.7303232	0.4057847	1	2.163.399
% ER patients referred to outpatient clinics	594	0.4666371	0.458148	0.2511887	0	0.28584489	0.6279859	0.3421410	1	2.412.181
% urgent surgeries	594	0.2723153	0.183609	0.2499626	0	0.09882245	0.3743688	0.2755463	1	4.170.949
% compliance with Surgical Checklist	594	0.5227962	0.501732	0.3286825	0	0.26654253	0.8190955	0.5525529	1	1.849.480
% Ebitda	594	0.6493975	0.708173	0.2509267	0	0.51126456	0.8350891	0.3238246	1	3.116.660
6 Month Profit Margin	594	0.5567577	0.586096	0.2680023	0	0.37772976	0.7587721	0.3810423	1	2.298.339
6 Month Profit Margin	594	0.5160802	0.516755	0.2811279	0	0.29663797	0.7476576	0.4510197	1	1.985.465
% ER Admissions	594	0.4469628	0.435100	0.2432254	0	0.26384919	0.5943219	0.3304727	1	2.653.205
% AnesthesiaConsent Comp	594	0.4983963	0.501858	0.3807310	0	0.07205624	0.8641484	0.7920922	1	1.426.135
% Surgery Consent Comp	594	0.4721966	0.414299	0.3618219	0	0.12868801	0.8169344	0.6882464	1	1.569.656
% OR Occupancy	594	0.6061732	0.645677	0.2399616	0	0.46097425	0.7808812	0.3199069	1	2.966.453
% New Outpatient Consult	594	0.3503227	0.298646	0.2541717	0	0.14703291	0.4948629	0.3478300	1	2.971.923
% First Consult Rate	594	0.4522047	0.433054	0.2482837	0	0.28615577	0.6152467	0.3290909	1	2.521.172
% ER Under 30Min	594	0.5229434	0.536613	0.2599961	0	0.34043865	0.7019140	0.3614753	1	2.297.122
% ER Under 90Min	594	0.6279091	0.674130	0.2505106	0	0.49036476	0.8111679	0.3208032	1	2.944.982
% Triaged ER Patients	594	0.5761908	0.616900	0.2784904	0	0.36978276	0.8106211	0.4408384	1	2.154.821
Inverse Comp Ratio	594	0.5583767	0.551634	0.2945026	0	0.33386076	0.7819565	0.4480958	1	2.026.492
Inverse First Wait	594	0.5125844	0.486766	0.2956755	0	0.29469806	0.7530959	0.4583978	1	1.931.598
Inverse Avg Stay Ratio	594	0.5750863	0.611111	0.2412639	0	0.42026316	0.7500000	0.3297368	1	2.762.760
Inverse Mortal Ratio	594	0.5753679	0.607941	0.2591283	0	0.41936572	0.7614213	0.3420556	1	2.596.616
Inverse Readmiss Ratio	594	0.5222858	0.544786	0.2627989	0	0.33050847	0.7185104	0.3880019	1	2.257.291
Medical Discharges	594	0.5272811	0.545841	0.2659106	0	0.32171659	0.7239978	0.4022812	1	2.189.476

Surgical Discharges	594	0.6159290	0.644489	0.2481880	0	0.49171135	0.8045194	0.3128080	1	3.070.504
Major Outpt Surg Ratio	594	0.4638759	0.440000	0.2514925	0	0.27832512	0.6363636	0.3580385	1	2.390.921
ComplexityRatio	594	0.5798319	0.615384	0.2527378	0	0.42857143	0.7777778	0.3492063	1	2.685.291
Complete Nursing Eval	594	0.4644458	0.461424	0.3230499	0	0.20000000	0.7222222	0.5222222	1	1.842.850
Available OR Hours	594	0.6374275	0.678966	0.2374572	0	0.54182788	0.7959441	0.2541163	1	3.881.747
Complete DischargeRpt	594	0.5247229	0.542746	0.3328526	0	0.28571429	0.8139535	0.5282392	1	1.824.304
Diagnosis Intens Ratio	594	0.4958065	0.488372	0.2775192	0	0.27906977	0.7185714	0.4395017	1	1.993.506
Complete Surgical Rpt	594	0.5487219	0.567738	0.3036424	0	0.32592593	0.7873070	0.4613811	1	2.127.629
NPS Outpatient Consult	594	0.5270794	0.541666	0.2509835	0	0.35100902	0.7142857	0.3632767	1	2.388.977
NPS Major Outpt	594	0.5500166	0.571428	0.2453221	0	0.37687387	0.7272727	0.3503989	1	2.513.398
Global NPS	594	0.5351918	0.549193	0.2448952	0	0.38333333	0.7149194	0.3315860	1	2.468.385
Nps Hospitalization	594	0.5373287	0.551512	0.2483154	0	0.37500000	0.7179487	0.3429487	1	2.421.218
Nps ER	594	0.5505968	0.567117	0.2528495	0	0.39247830	0.7297297	0.3372514	1	2.427.809
Total Surgeries	594	0.6169986	0.643975	0.2343563	0	0.52813062	0.7713792	0.2432486	1	3.648.200
Total Births	594	0.3763869	0.380597	0.2961687	0	0.00000000	0.5994141	0.5994141	1	1.951.191
High Complex Tech	594	0.5430798	0.565784	0.2515450	0	0.37180698	0.7237541	0.3519472	1	2.472.843
Bed Occupancy	594	0.5829314	0.607142	0.2363940	0	0.42857143	0.7427489	0.3141775	1	2.910.226
First Medical Visits	594	0.5978129	0.631084	0.2430004	0	0.46445336	0.7670073	0.3025540	1	2.981.481
First Surgical Visits	594	0.5702289	0.588696	0.2476973	0	0.40542063	0.7511126	0.3456920	1	2.566.317
Adult ER Visits	594	0.4416412	0.436428	0.2310488	0	0.28537626	0.5897456	0.3043693	1	2.842.708
Pediatric ER Visits	594	0.4611832	0.451916	0.2891285	0	0.22372578	0.6837640	0.4600382	1	1.976.608
Sales Value	594	0.5269580	0.553207	0.2748325	0	0.34123419	0.7365584	0.3953242	1	2.145.568
3 Month Sales Value	594	0.5606034	0.578434	0.2741816	0	0.35622017	0.7843100	0.4280898	1	2.088.817
6 Month Sales Value	594	0.4493991	0.448544	0.2842534	0	0.20079794	0.6748300	0.4740321	1	1.926.891
Complete Med Orders	594	0.5884389	0.628151	0.3565718	0	0.26692088	10.000.000	0.7330791	1	1.622.537
