# Supplementary material for: Exploring the Interplay Between Healthcare Quality and Economic Viability Through Massive Data Analysis-Driven Multi-Hospital Management in a Spanish Private Multi-Hospital Network
Source: Healthcare (Basel). 2025 Nov 24;13(23):3034. doi: 10.3390/healthcare13233034 (PMC12692472; doi:10.3390/healthcare13233034)
Supplement: Supplementary file 1 [file healthcare-13-03034-s001.zip › Supplementary Table S2.pdf]

Supplementary Table S2*. Table clusters – Indicators kmeans.*

**Cl**	**KPIs**
**1**	(E) % ER To Outpacient Rate, (E) % ERAdmissions, (E) % ERUnder30Min , (E) % Triag ed ERPatients, (E) Global NPS, (E) InverseMortalRatio, (C) InverseReadmissRatio , (NG) % SurgeryConsentComp , (NG) InverseAvgStayRatio, (NG) NPS ER
**2**	(F) % AnesthesiaConsentComp, (F) % Early Disch Before Noon, (F) CompleteNursingE val, (F) CompleteSurgicalRpt, (F) DiagnosisIntensRatio, (F) InverseFirstWait
**3**	(B) CompleteDischargeRpt, (B) CompleteMedOrders, (B) NPS Hospitalization, (C) % S urgCheckCompliance, (D) AdultERVisits, (D) PediatricERVisits
**4**	(NG) % NewOutpatientConsult , (NG) % Urgent Surgeries
**5**	(C) MajorOutptSurgRatio, (C) NPSMajorOutptSurg, (C) NPSOutpatientConsult, (NG) % First Consult Rate , (NG) InverseCompRatio
**6**	(A) % OR Occupancy, (A) AvailableORHours , (A) Bed Occupancy, (A) ComplexityRat io, (A) First Medical Visits, (A) First Surgical Visits, (A) HighComplexTech, (A) S urgical Discharges, (A) TotalBirths, (A) TotalSurgeries, (D) Medical Discharges, (NG) % ERUnder90Min

Table S2. This table presents the results of a k-means clustering analysis, where the key performance indicators (KPIs) have been organized into six distinct groups (Cluster 1 to 6). The Cluster column identifies the numerical group, and the Indicators column lists the KPIs that comprise each group. Each KPI is preceded by a letter in parentheses, likely denoting its domain or category (e.g., E, F, B, C, D, A, NG).
